# The complete conformational free energy landscape of β-xylose reveals a two-fold catalytic itinerary for β-xylanases†
†Electronic supplementary information (ESI) available. See DOI: 10.1039/c4sc02240h



**DOI:** 10.1039/c4sc02240h

**Published:** 2014-10-27

**Authors:** Javier Iglesias-Fernández, Lluís Raich, Albert Ardèvol, Carme Rovira

**Affiliations:** a Departament de Química Orgànica and Institut de Química Teòrica i Computacional (IQTCUB) , Universitat de Barcelona , Martí i Franquès 1 , 08028 Barcelona , Spain . Email: c.rovira@ub.edu; b Department of Chemistry and Applied Biosciences , ETH Zürich , USI Campus , 6900 Lugano , Switzerland; c Institució Catalana de Recerca i Estudis Avançats (ICREA) , Passeig Lluís Companys , 23 , 08018 Barcelona , Spain

## Abstract

Unraveling the conformational catalytic itinerary of glycoside hydrolases (GHs) is a growing topic of interest in glycobiology, with major impact in the design of GH inhibitors. β-xylanases are responsible for the hydrolysis of glycosidic bonds in β-xylans, a group of hemicelluloses of high biotechnological interest that are found in plant cell walls. The precise conformations followed by the substrate during catalysis in β-xylanases have not been unambiguously resolved, with three different pathways being proposed from structural analyses. In this work, we compute the conformational free energy landscape (FEL) of β-xylose to predict the most likely catalytic itineraries followed by β-xylanases. The calculations are performed by means of *ab initio* metadynamics, using the Cremer–Pople puckering coordinates as collective variables. The computed FEL supports only two of the previously proposed itineraries, ^2^S_O_ → [^2,5^B]^ǂ^ → ^5^S_1_ and ^1^S_3_ → [^4^H_3_]^ǂ^ → ^4^C_1_, which clearly appear in low energy regions of the FEL. Consistently, ^2^S_O_ and ^1^S_3_ are conformations preactivated for catalysis in terms of free energy/anomeric charge and bond distances. The results however exclude the ^O^E → [^O^S_2_]^ǂ^ → B_2,5_ itinerary that has been recently proposed for a family 11 xylanase. Classical and *ab initio* QM/MM molecular dynamics simulations reveal that, in this case, the observed ^O^E conformation has been enforced by enzyme mutation. These results add a word of caution on using modified enzymes to inform on catalytic conformational itineraries of glycoside hydrolases.

## Abstract 2

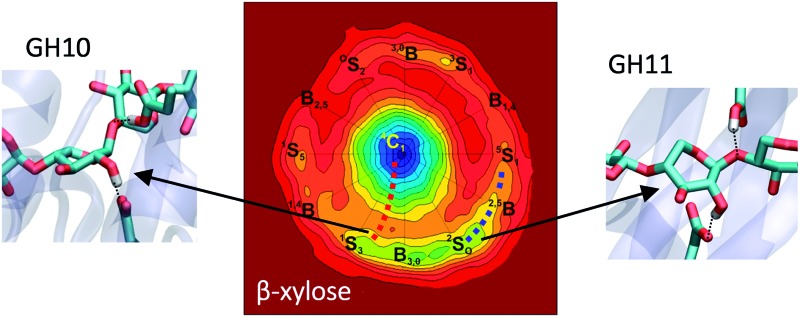
 *Ab initio* conformational free energy landscapes, together with molecular dynamics simulations, enable to predict the catalytic itineraries of β-xylanase enzymes.

## Introduction

Carbohydrates are the most abundant biomolecules on Earth. They have a huge diversity of roles, ranging from structural elements in the cell walls of bacteria and plants to cell–cell recognition processes of nonphotosynthetic cells. The vast amount and diversity of carbohydrate-based structures present in nature requires a large group of enzymes responsible for their metabolism.^1^ This is the function of glycoside hydrolases (GHs), polysaccharide lyases (PLs) or glycosyltransferases (GTs), which constitute approximately 1–2% of the genome of any organism.^2,3^

GHs are highly specific enzymes that catalyze the cleavage of glycosidic bonds in carbohydrates. These enzymes are systematically classified on the basis of amino acid sequence similarity into 133 families (see http://www.cazy.org and ; http://www.cazypedia.org),^4^ which generally share a common fold and reaction mechanism: retention or inversion of the anomeric configuration. With some exceptions,^5^ retaining GHs follow a double displacement mechanism, with the formation of a covalent glycosyl–enzyme intermediate, whereas inverting GHs operate on one unique step.^6^ Regardless of the mechanism, the transition state of the reaction has oxocarbenium ion-like character.^6^

**Fig. 1:**
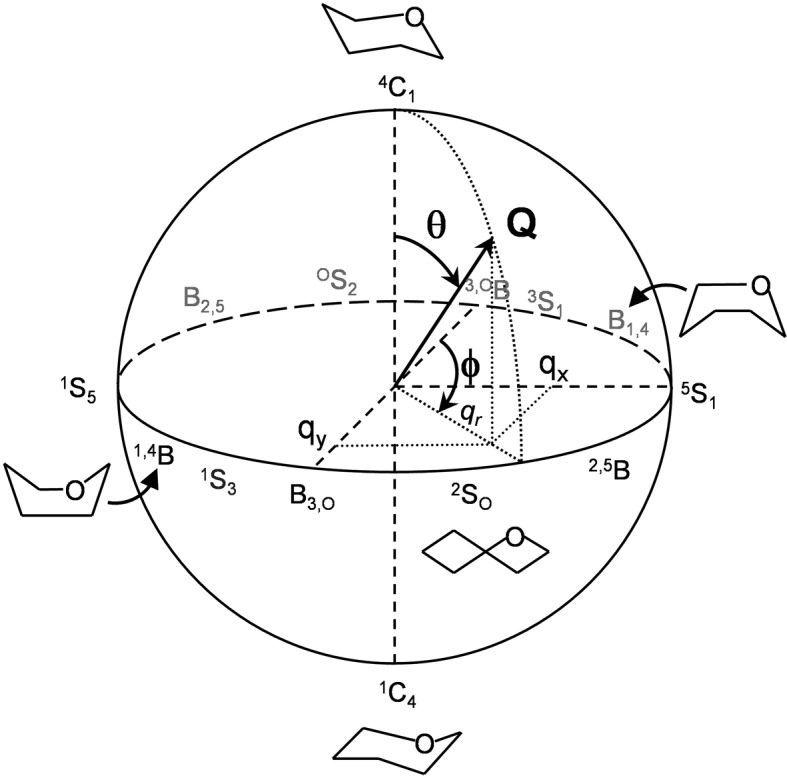
Cremer and Pople puckering coordinates of a six-membered ring (*Q*, *θ*, and *φ*) and their projection in the *x*, *y* plane (*q*_*x*_ and *q*_*y*_).

**Fig. 2:**
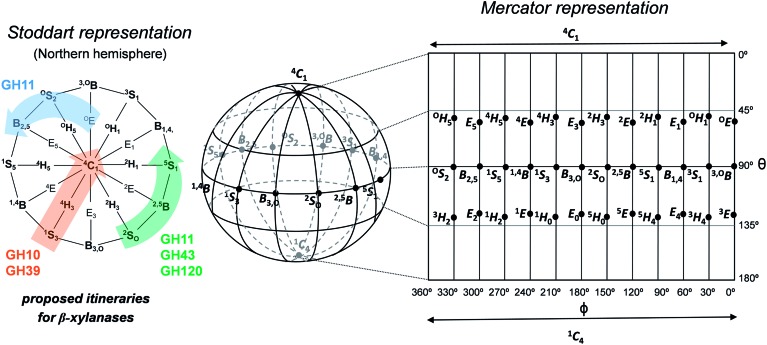
Main conformations on the Cremer–Pople sphere along with two of their most used representations: Stoddart (Northern projection) and Mercator. Proposed itineraries for β-xylanases belonging to several GH families are depicted on Stoddart diagram.

It is nowadays well established that the carbohydrate substrate undergoes critical conformational changes upon binding to the GH active site.^7,8^ As observed in NMR and X-ray experiments,^9–11^ the sugar ring located at the –1 enzyme subsite distorts away from its ^4^C_1_ conformation in solution towards a high-energy one (*e.g.*, boat or skew-boat) upon binding to GHs. A fascinating thread of GH research, and one with major impact on the design of enzyme inhibitors, is the conformational analysis of reaction pathways within the diverse enzyme families.^7,8,12^ Different enzyme families can harness different reaction pathways and, therefore, transition states. In general, GHs with a common type of mechanism (retention or inversion of the anomeric configuration) that act on similar substrates follow a common catalytic itinerary.^7,8^ For instance, retaining β-glucosidases follow a ^1^S_3_ → [^4^H_3_]^ǂ^ → ^4^C_1_ itinerary, whereas inverting β-glucosidases use ^2^S_O_ → [^2,5^B]^ǂ^ → ^5^S_1_. These itineraries can be drawn on the Cremer–Pople^13^ puckering sphere (a representation of all possible ring conformations defined by the puckering or polar coordinates *Q*, *φ* and *θ*; see Fig. 1).^14^ However, simpler 2D projections, such as Stoddart or Mercator diagrams, are normally used to draw catalytic itineraries (Fig. 2).^8,15^

β-xylanases, responsible for the hydrolysis of glycosidic bonds in β-xylans, are particularly interesting for their wide range of industrial applications, such as bioenergy and biofuel production from plant cell-wall biomass, in bread production, in pulp and paper industry and in the enhancement of animal feedstocks.^16–18^ An intriguing aspect of β-xylanases is that, unlike other types of GHs, they have been proposed to follow several conformational itineraries (Fig. 2). In the case of retaining β-xylanases, three itineraries have been predicted: one that involves a ^4^H_3_ transition state (^1^S_3_ → [^4^H_3_]^ǂ^ → ^4^C_1_), a second one that passes through a ^2,5^B transition state (^2^S_O_ → [^2,5^B]^ǂ^ → ^5^S_1_) and a third one that involves an unusual ^O^S_2_ transition state (^O^E → [^O^S_2_]^ǂ^ → B_2,5_). Support for a ^1^S_3_ → [^4^H_3_]^ǂ^ → ^4^C_1_ itinerary is based on the observation of covalently bound substrates (2-deoxy-2-fluoroxylopyranosyl substrate) exhibiting a ^4^C_1_ conformation in a family 10 GH^19,20^ and ^1^S_3_ conformation for the Michaelis complex of another GH10 xylanase.^21^ In addition, xylobio-imidazole and xylobio-lactam oxime TS-like inhibitors have been found to adopt conformations close to ^4^H_3_ (^4^E) in a GH10 xylanase^22^ (this is particularly relevant in the light of a recent work on mannosidase inhibitors showing that imidazole derivatives exhibit good TS shape mimic properties).^23^ Experiments on a GH39 xylanase also agree with a ^1^S_3_ → [^4^H_3_]^ǂ^ → ^4^C_1_ itinerary.^24^ Support for the second itinerary (^2^S_O_ → [^2,5^B]^ǂ^ → ^5^S_1_) mainly originates from reports on covalently bound substrates of family 11 GHs in which the xylose saccharide located at the –1 subsite (2-deoxy-2-fluoroxylopyranosyl substrate) adopts a ^2,5^B conformation,^25,26^ as well as experiments on synthetic xyloside analogues locked on a ^2,5^B conformation.^27^ Computational modeling studies on *B. circulans* GH11 xylanase addressed this question, concluding that the conformation of the xylose saccharide in the Michaelis complex is either ^2^S_O_ or ^2,5^B.^28,29^ To add to the confusion, a recent structural study reports an almost undistorted conformation (^4^C_1_/^O^E) for a xylohexaose substrate in complex with a GH11 xylanase mutant, predicting an unusual ^O^E → [^O^S_2_]^ǂ^ → B_2,5_ itinerary (Fig. 2).^30^ This is particularly surprising since the ^O^S_2_ conformation does not fulfill the stereochemical requirements of an oxocarbenium ion-like transition state (ideally, C5, O5, C1 and C2 atoms should be coplanar). Finally, inverting β-xylanases are expected to follow ^2^S_O_ → [^2,5^B]^ǂ^ → ^5^S_1_ itinerary,^31,32^ although only family 43 has been so far characterized. Therefore, the precise conformations followed by the substrate during catalysis in β-xylanases, especially those of family 11, have not been unambiguously resolved.

Detailed information concerning the energetic, structural and electronic relations of xylose is necessary for the understanding of xylosidase reaction mechanisms, especially to elucidate whether several catalytic itineraries for β-xylanases are possible. It has been previously shown that the conformational free energy landscape (FEL) of isolated simple sugars, obtained by *ab initio* metadynamics, informs about the conformations being preactivated for catalysis in GHs acting on the given sugar (*e.g.* β-glucose in β-glucosidases, β-mannose for β-mannosidases, *etc.*) and, therefore, can be used to predict catalytic conformational itineraries.^8^ Recent metadynamics studies using force-fields have contributed to popularize the representation of sugar conformational maps,^33,34^ in the spirit of the seminal work by Dowd, French and Reilly.^35,36^ Pederiva *et al.* ^37–39^ raised controversy about the influence of the choice of the collective variables (*i.e.* Cartesian coordinates, *q*_*x*_ and *q*_*y*_, or polar coordinates, *θ* and *φ*; see Fig. 1) in metadynamics simulations of sugar puckering. In addition, several works on six-membered ring conformations have also appeared in recent years (see *e.g.* ref. 40). Of particular interest is the study of Mayes *et al.*, in which several six-membered rings are studied using standard optimization techniques within Density Functional Theory (DFT).^41^ Unfortunately, the relevant transition between ^1^S_3_ and ^4^C_1_ in β-xylose, which is one of the main pathways proposed experimentally for β-xylosidases, was not resolved. Therefore, a complete free energy landscape for β-xylose is still missing.

In this work, we first demonstrate that the conformational free energy landscape obtained by *ab initio* metadynamics is invariant with respect of the type of collective variables (polar or Cartesian) used in the calculations. In a second step, we provide the FEL of β-xylose and use it to assess the catalytic itineraries that have been proposed for retaining β-xylosidases. Finally, we perform classical and quantum mechanics/molecular mechanics (QM/MM) molecular dynamics (MD) simulations on two selected E·S complexes to evaluate the effect of enzyme mutation on substrate conformation.

## Computational details

### Stoddart and Mercator representations of the puckering sphere

Any of the possible conformations of a six-atom sugar ring can be unequivocally assigned using the 3 Cremer and Pople puckering coordinates *Q*, *φ* and *θ* (Fig. 1).^13^ The *Q* coordinate is the sum of the perpendicular distance of each ring atom (*j*) to the ring average plane 
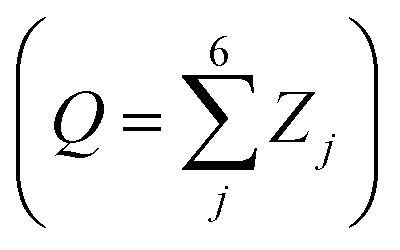
. The *φ* and *θ* coordinates are obtained by solving the following system of equations.
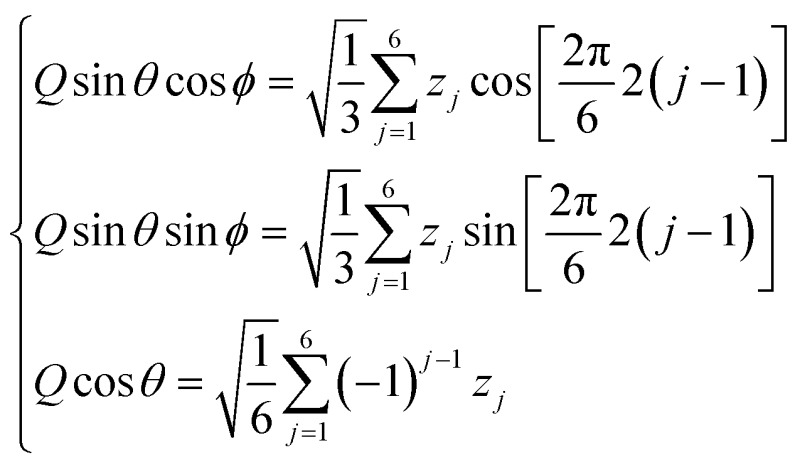


Since these are polar coordinates, any ring distortion would fall within the puckering sphere-like volume (Fig. 1). While *Q* may differ among different conformations, *θ* and *φ* are sufficient to differentiate between all the conformers of Fig. 1. On the poles (*θ* = 0 or π) are located the two chair conformers (^4^C_1_ and ^1^C_4_, respectively); on the equatorial region (*θ* = π/2) the 6 boat and 6 skew-boat structures are sequentially placed in steps of *φ* = π/6.

Two representations have been historically used to map the Cremer and Pople 3D plot into a simpler two dimensional plot. Stoddart diagram^42^ corresponds to the projection of the polar coordinates onto the equatorial plane and can be defined by the Cartesian coordinates *q*_*x*_ and *q*_*y*_ (Fig. 2).^43^ On another hand, the so-called plate carrée or Mercator representation is an equidistant cylindrical projection that results in a rectangular map with respect to *θ* and *φ*.^35^ Which representation to use is essentially a matter of choice. Catalytic GH itineraries are easy to interpret when they are drawn on Stoddart representation, as there are no discontinuities between conformations. Moreover, since GHs normally follow straight itineraries either on the Northern or Southern hemispheres, but not on both (*i.e.* they do not follow transitions from ^4^C_1_ to ^1^C_4_ or *vice versa*), only one diagram is sufficient to sample the experimentally relevant conformations, which further simplifies the problem. On another hand, the Mercator representation represents all conformations on a single diagram, thus mapping the complete Cremer–Pople sphere. This can be most convenient in a more general context, if one is interested in sampling all conformations of the sugar ring.

### *Ab initio* molecular dynamics simulations

All the first principles simulations at room temperature reported in this work were performed within the Car–Parrinello approach,^44^ as implemented in the CPMD 3.15.1 program.^45^ The analyzed systems consist of a single cyclohexane or β-xylose unit enclosed in an orthorhombic box of size 11 Å × 11 Å × 11.5 Å and 12.5 Å × 13.5 Å × 11.6 Å, respectively. The electronic structure was computed within the DFT, using the Perdew, Burke, and Ernzerhoff generalized gradient-corrected approximation (PBE).^46^ This functional gave reliable results in previous Car–Parrinello simulations of isolated carbohydrates and GHs. In particular, the error on relative energies due to the DFT functional employed is ±0.6 kcal mol^–1^ for β-glucose.^43^ Kohn–Sham orbitals were expanded in a plane wave (PW) basis set with a kinetic energy cutoff of 70 Ry. Norm-conserving pseudopotentials were employed, generated within the Troullier–Martins scheme.^47^ The fictitious mass for the electronic degrees of freedom was set to 1000 au and 850 au for the cyclohexane and xylose systems, respectively, as used in previous studies of isolated sugars,^43,48^ β-glucose in solution^49^ and carbohydrate-active enzymes.^32,50–53^

### Metadynamics simulations

Metadynamics (MTD) is a molecular dynamics based technique that enables the phase space exploration and the estimation of the FEL. This method is based on the reduction of the phase space dimensionality to a smaller set of so-called “collective variables” (CVs) which enclose the slowest modes that are relevant to the process of interest. The addition of small repulsive potentials on this reduced phase space during the molecular dynamics simulation enhances the exploration of the phase space. When convergence is reached, the inverse of the sum of the added potentials corresponds to the underlying free energy profile on the CVs space.^54^ A detailed explanation of the method can be found elsewhere.^55^ Metadynamics was used to sample all conformations of the puckering sphere of the 6-membered rings analyzed here (cyclohexane and β-xylose) and obtain their conformational free energy landscapes.

The representation of the FEL with respect to the Mercator diagram (“Mercator FEL”) was obtained using directly the *φ* and *θ* polar puckering coordinates as collective variables in the metadynamics simulation. On another hand, the “Stoddard FEL” was obtained from the Cartesian coordinates *q*_*x*_ and *q*_*y*_ (Fig. 2), defined as *Q* sin(*θ*)sin(*φ*) and *Q* sin(*θ*)cos(*φ*), respectively. Both types of collective variables (polar and Cartesian) were used for the simulations of cyclohexane, whereas only polar coordinates were used for β-xylose.

The height of the Gaussian terms was set to 0.13 kcal mol^–1^ (cyclohexane) and 0.18 kcal mol^–1^ (xylose), which ensures sufficient accuracy for the reconstruction of the FEL. The width of the Gaussian terms was set to 0.1 CV units in both cases, according to the oscillations of the selected collective variables observed in the free dynamics. The extended Car–Parrinello Lagrangian was used to describe the dynamics of the collective variables for the simulation using *q*_*x*_/*q*_*y*_ variables, as done in our previous work.^43,48^ The mass of the fictitious particle and the force constant of the coupling potential were tested to ensure that the coupled particle follows the value of the associated collective variable in the real system. Values of 5.0 amu for the mass of the fictitious particle and 0.5 au for the force constant fulfill these conditions. A direct implementation of the MTD algorithm was used to obtain the Mercator FEL. A new Gaussian potential was added every 200–400 MD steps at the first stage of the simulation, increasing it up to 1000 MD steps to ensure a proper convergence of the simulation. A total number of 3500/9000 Gaussian functions were added to completely explore the free energy landscape of cyclohexane (Mercator/Stoddart representations) and 4500 for β-xylose (Mercator representation). The convergence of the metadynamics simulations was assessed by checking the invariance of energy differences and the free energy landscape with the progression of the simulation, following ref. 56. The convergence error in the energies was found to be lower than 0.5 kcal mol^–1^.

### Modeling of E·S complexes

Classical MD and *ab initio* QM/MM MD simulations were performed to test the effect of enzyme mutations on the Michaelis complex of *Trichoderma ressei* GH11 xylanase in complex with xylohexaose (PDB entry ; 4HK8),^30^ as well as the Michaelis complex of *Streptomyces olivaceoviridis* GH10 xylanase in complex with xylopentaose (PDB entry ; 2D24).^21^ The AMBER^57^ and CPMD^45^ codes were used for the classical MD and *ab initio* QM/MM simulations,^58^ respectively. The set up of the systems and simulation details are reported in the ESI (Sections 4 and 5†).

## Results and discussion

### The conformational free energy landscape of cyclohexane

Cyclohexane, the most simple six-membered ring for which experimental information of the relative energy among conformations is available, was used to test the dependence of the conformational free energy landscape with respect of the type of collective variables used in the metadynamics simulation (polar variables, *θ* and *φ*, leading to the Mercator representation, or Cartesian variables, *q*_*x*_ and *q*_*y*_, leading to the Stoddart representation).

**Fig. 3:**
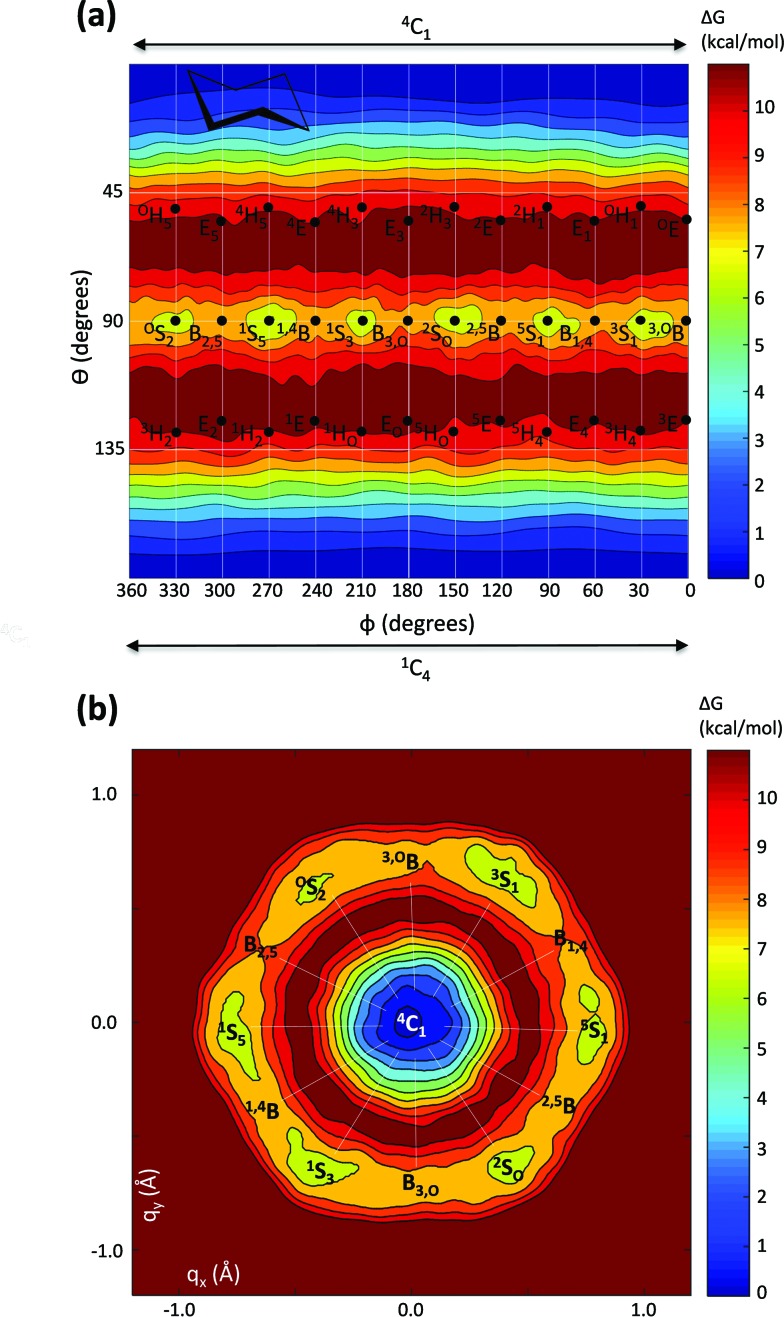
(a) Computed free energy landscape of cyclohexane resulting from the first principles metadynamics using *θ* and *φ* as collective variables (Mercator representation). The continuous lines, separated by 30° in *φ*, indicate regions corresponding to different canonical conformations. (b) Computed free energy landscape of cyclohexane resulting from the *ab initio* metadynamics using *q*_*x*_ and *q*_*y*_ as collective variables (Stoddart representation). Energy values are given in kcal mol^–1^ and each contour line of the diagram corresponds to 1 kcal mol^–1^.

The conformational free energy landscape of cyclohexane, reconstructed from the metadynamics simulation using *θ* and *φ* as collective variables (*i.e.* Mercator representation), is shown in Fig. 3a. The most stable conformers are ^4^C_1_ and ^1^C_4_, which are equivalent.^59^ The skew-boat conformers correspond to local minima, 6.3 kcal mol^–1^ higher in energy than the chair conformers, whereas boat conformations lay 1 kcal mol^–1^ higher in energy and correspond to transition states between skew-boats. Interconversion from chair to any skew-boat crosses a half-chair conformer with an energy barrier of 10.5 kcal mol^–1^. Half-chair and envelope conformations are energetically equivalent. These values are in good agreement with experimental estimates (Δ*G*chair→skew-boat = 10.4–10.8 kcal mol^–1^; Δ*G*_chair↔skew-boat_ = 4.7–6.2 kcal mol^–1^),^60–62^ as well as previous theoretical predictions (see for instance ref. 40). It is noteworthy that the transition states (TS) between skew-boat and chair conformers occur at *θ* angles of 60° (Northern hemisphere) and 120° (Southern hemisphere), thus they deviate from pure half-chair conformations. The last are located at 50.8° and 129.2°, respectively, and display four consecutive coplanar atoms.^13^ A separate analysis by standard TS search methods using various levels of theory (page S2 of the ESI†) leads to similar results. The non-planarity of the transition state, already observed by Hendrickson in 1967,^63^ has often been overlooked in the literature.^64,65^

The FEL obtained by using Cartesian coordinates (*q*_*x*_ and *q*_*y*_) as collective variables is shown in Fig. 3b. Similarly to the Mercator FEL, it reflects the expected hexagonal symmetry, within statistical errors due to finite sampling of the phase space.^66^ Remarkably, it exhibits the same features as the simulation using polar coordinates as collective variables, resulting in very similar free energy differences (Δ*G*chair→skew-boat = 10.5 kcal mol^–1^ and Δ*G*_chair↔skew-boat_ = 6.0 kcal mol^–1^). These results demonstrate that metadynamics simulations performed either with polar or Cartesian coordinates give equivalent results.

**Fig. 4:**
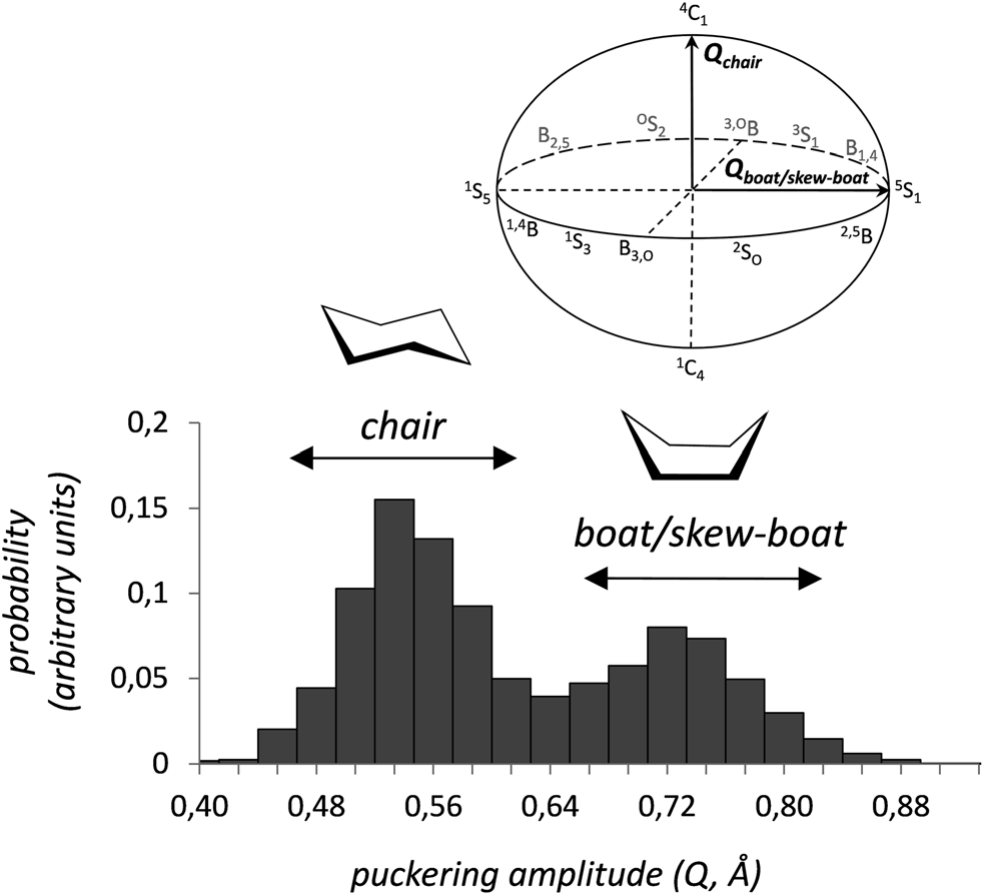
Probability distribution for the radial coordinate *Q* in cyclohexane.

It is interesting to analyze not only the variation of the *θ* and *φ* coordinates, which determine ring conformation, but also the puckering amplitude *Q* (Fig. 1), which gives an idea of the degree of “puckering” of the ring. Analysis of the structures obtained in the metadynamics simulation evidences that the distribution of *Q* values, *P*(*Q*), features a bimodal behavior (Fig. 4), with peaks around 0.54 Å and 0.72 Å for the chair and skew-boat conformations, respectively. Therefore, the puckering volume is not spherical but ellipsoidal, with the *Q* radii increasing slowly from the poles to the equator.

The bimodal distribution of the puckering amplitude contrast with the results obtained in a previous study using force-fields,^37^ which show a unique narrow peak around 0.5 Å for the probability distribution. This type of unimodal distribution would lead to anomalous values of the <CCC angles, creating substantial ring strain on the boat and skew-boat conformations. In the above mentioned work,^37^ it was also argued that calculations using the Cartesian coordinates *q*_*x*_ and *q*_*y*_ can affect the range of sampled conformations at different *Q*. This conclusion was based on the plot of the puckering amplitude *Q vs. q*_*r*_, where 
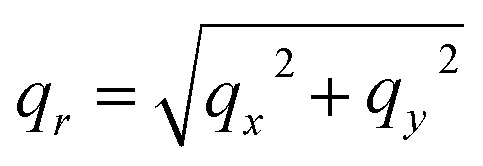
 is the projected puckered vector (see Fig. 1), which shows a strong correlation for *q*_*r*_ values larger than 0.5 Å. However, as it is shown in Fig. S2,† the two metadynamics simulations performed in this work display a very similar dual correlation between *q*_*r*_ and *Q*, which is consistent with the puckering amplitude displaying a bimodal distribution. Therefore, both Cartesian and polar CVs lead to a similar correlation between *Q* and *q*_*r*_.^67^

### The conformational free energy landscape of β-xylose

**Fig. 5:**
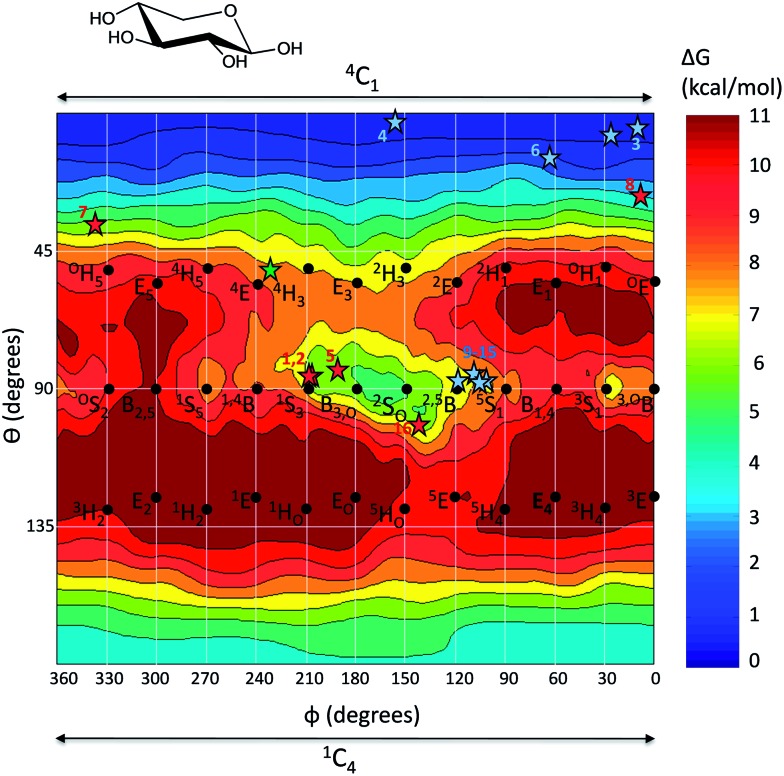
Computed free energy landscape of β-xylose (Mercator representation) with respect to ring distortion. Energy values are given in kcal mol^–1^ and each contour line of the diagram corresponds to 1 kcal mol^–1^. The conformations found in experimental structures of retaining β-xylosidases are represented by red color stars (Michaelis complex structures) and blue color stars (covalent intermediate structures). The conformation of the TS-like inhibitor xylobio-imidazole in complex of family 10 xylanase Cex from *Cellulomonas fimi* ^22^ has also been indicated (green color star). For the sake of clarity, only one star is displayed for several structures with nearly identical conformations.

The free energy landscape of β-d-xylose in the Mercator representation is shown in Fig. 5. It contains several local minima. Two of them at the poles, corresponding to the two chair conformers (^4^C_1_ and ^1^C_4_, at *θ* = 0° and 180°, respectively), as well as a two local minima in the equator (*θ* = 90°): a wide minimum centered at ^2^S_O_/B_3,O_ (*φ* = 150–180°) and a small minimum centered at ^3^S_1_/^3,O^B (*φ* = 0–30°). As previously observed for other sugar molecules,^43,48^ not all minima have a direct correspondence with the ideal conformations of the Mercator diagram. For instance, conformations B_1,4_ and B_2,5_ do not correspond to energy minima but lay in high energy regions of the FEL. On another hand, there are minima in between two canonical conformations (*e.g.* ^2^S_O_/B_3,O_).

It is clear from Fig. 5 that the ^4^C_1_ chair is the global minimum. The inverted chair, ^1^C_4_, is 4 kcal mol^–1^ higher in energy, closely followed by the mixed ^2^S_O_/B_3,O_ conformation, which is 5 kcal mol^–1^ above ^4^C_1_. The remaining minima (^3,O^B/^3^S_1_, ^1^S_5_, ^5^S_1_) are much higher in energy (≥7 kcal mol^–1^). A noticeable feature of the FEL is the presence of a wide valley on the equator, covering conformations ^1^S_3_–B_3,O_–^2^S_O_–^2,5^B–^5^S_1_, which is connected with the ^4^C_1_ global minimum by a low energy region at *θ* = 75°.

One might wonder whether the particular shape of the FEL is related with the occurrence of distorted conformations in β-xylosidase complexes. To this aim, we obtained the puckering coordinates of the sugar ring at the –1 subsite in all available structures of β-xylosidase complexes (Table 1), either Michaelis complexes (E·S) or glycosyl–enzyme covalent intermediates. Most of these complexes were obtained from enzyme mutants. It turned out that all experimental structures of Michaelis complexes (Fig. 5) and their corresponding TS predicted structures are located in low-energy regions of the free energy landscape. For instance, structures 1–2 and 5 (Table 1) correspond to complexes of GH10 and GH39 β-xylosidases, for which a ^4^H_3_ transition state is expected. Even though none of the experimental points and the predicted TS lie precisely at the center of a FEL minimum, they fall into a low energy region of the FEL. Structure 7 (see Table 1) is a Michaelis complex of a GH11 xylanase. Structure 8, corresponding to a glutamine mutant of the acid/base residue, is anomalous, as it features a –1 xylose saccharide in a practically undistorted conformation, which is intermediate between ^4^C_1_ and ^O^E (the origin of this peculiar conformation will be discussed later on). Structures 9–15 (see Table 1 and blue stars near ^2,5^B), correspond to covalent intermediates of the hydrolysis reaction of GH11 β-xylosidases, for which a ^2,5^B transition state has been proposed. Structure 16 is a Michaelis complex of a GH120 β-xylanase from *Thermoanaerobacterium saccharolyticum*. Overall, Michaelis complex conformations and predicted transition states fall in low energy regions of the FEL, whereas there are no experimental structures in high energy regions. Therefore, we can conclude that the calculations of the isolated sugar molecules reveal lower free energy regions that agree with the possible conformational routes followed by different enzyme families. This does not mean, of course, that a given particular enzyme environment will not influence the conformational landscape reported here. In fact, it has been shown for an α-mannosidase^15^ and a β-glucosidase^51^ that the enzyme further restricts the accessible conformations of the sugar residue with respect to the ones observed in the gas phase. However, most of the conformations accessible on-enzyme are already reflected on the FEL of the isolated molecule, which can be considered as a fingerprint of the given sugar.

**Table 1:** Complexes of retaining β-xylanases, as well as the conformations adopted by the saccharide at subsite –1

Structure	PDB code	Family	Resolution (Å)	Substrate conformation	*θ*, *φ* (degrees)	Enzyme form	Type of complex^*a*^	Substrate^*b*^	Reference
1	2D20	GH10	1.85	^1^S_3_	87, 210	N127S, E128H	M. C.	pNP-Xyl_2_	21
2	2D24	GH10	1.85	^1^S_3_	87, 204	N127S, E128H	M. C.	Xyl_5_	21
3	2Xyl	GH10	1.90	^4^C_1_	5, 13	WT^*c*^	C. I.	2-deoxy-2F-Xyl_2_	19
4	2D22	GH10	1.70	^4^C_1_	3, 156	N127S, E128H	C. I.	Xyl_2_	21
5	2BFG	GH39	2.40	^1^S_3_	83, 191	E160A	M. C.	2,5-dNP-Xyl	24
6	2BFG	GH39	2.40	^4^C_1_	15, 63	E160A	C. I.	Xyl	24
7	2QZ3	GH11	1.80	^2^S_O_ ^*d*^	37, 337	E172A	M. C.	Xyl_3_	71
8	4HK8	GH11	1.15	^4^C_1_/^O^E	28, 9	E177Q	M. C.	Xyl_6_	30
9	1BVV	GH11	1.80	^2,5^B	85, 110	WT	C. I.	2-deoxy-2F-Xyl	26
10	1QH6	GH11	2.00	^2,5^B	88, 119	WT	C. I.	2-deoxy-2F-Xyl_2_	25
11	3ZSE	GH11	1.78	^2,5^B	89, 108	WT	C. I.	2-deoxy-2F-Xyl_2_	72
12	1C5I	GH11	1.80	^2,5^B	85, 108	N35D	C. I.	2-deoxy-2F-Xyl_2_	73
13	1H4G	GH11	1.10	^2,5^B	89, 117	WT	C. I.	2-deoxy-2F-Xyl_2_	25
14	3VZN	GH11	1.67	^2,5^B	84, 109	N35E	C. I.	2-deoxy-2F-Xyl_2_	74
15	3VZO	GH11	1.73	^2,5^B	88, 109	N35H	C. I.	2-deoxy-2F-Xyl_2_	74
16	3VSU	GH120	2.05	^2^S_O_	103, 141	WT	M. C.	Xyl_2_	75

^*a*^M. C. = Michaelis complex, C. I. = covalent intermediate.

^*b*^pNP = paranitrophenyl, dNP = dinitrophenyl.

^*c*^WT = wild type enzyme.

^*d*^Reported as ^2^S_O_ in ref. 71 but present as ^4^C_1_/^O^H_5_ in the deposited structure (monomer B).

**Fig. 6:**
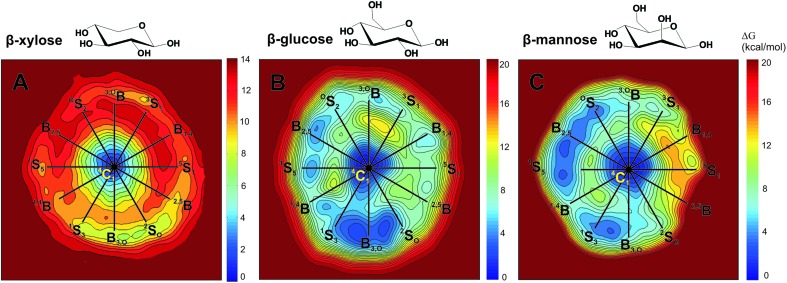
Conformational free energy landscapes (Stoddart representation of the puckering sphere Northern hemisphere) obtained for β-xylose (this work), β-glucose (ref. 43) and β-mannose (ref. 48). Each contour line of the diagram corresponds to 1 kcal mol^–1^ (β-xylose) and 0.5 kcal mol^–1^ (β-glucose and β-mannose).

As mentioned before, catalytic conformational itineraries have been often drawn with respect of Stoddart diagram (Fig. 2, left panel),^7,68^ in which all conformations are interconnected. Therefore, we transformed the Mercator map of Fig. 5 into a Stoddart representation (Fig. 6a)^69^ to identify conformational itineraries. It is easy to see that two of the itineraries that have been experimentally proposed (^1^S_3_ → [^4^H_3_]^ǂ^ → ^4^C_1_ and ^2^S_O_ → [^2,5^B]^ǂ^ → ^5^S_1_) cover the low energy regions of the FEL. In contrast, the ^O^E → [^O^S_2_]^ǂ^ → B_2,5_ itinerary, recently proposed for *Trichoderma ressei* GH11 xylanase, passes through a high energy region of the FEL. In the light of this and our previous studies on isolated sugars (β-d-glucose,^43^ α- and β-d-mannose,^15,48^ α-l-fucose^70^) this is unprecedented and it might indicate that trends derived from the computed FEL break down in this case. However, the fact that the ^O^S_2_ conformation proposed as TS does not fulfill the stereochemical requirements of an oxocarbenium ion-like transition state (C5, O5, C1 and C2 atoms should be coplanar) suggests that the complex obtained with the modified enzyme might correspond to a non-productive complex (or a pre-Michaelis complex).

**Fig. 7:**
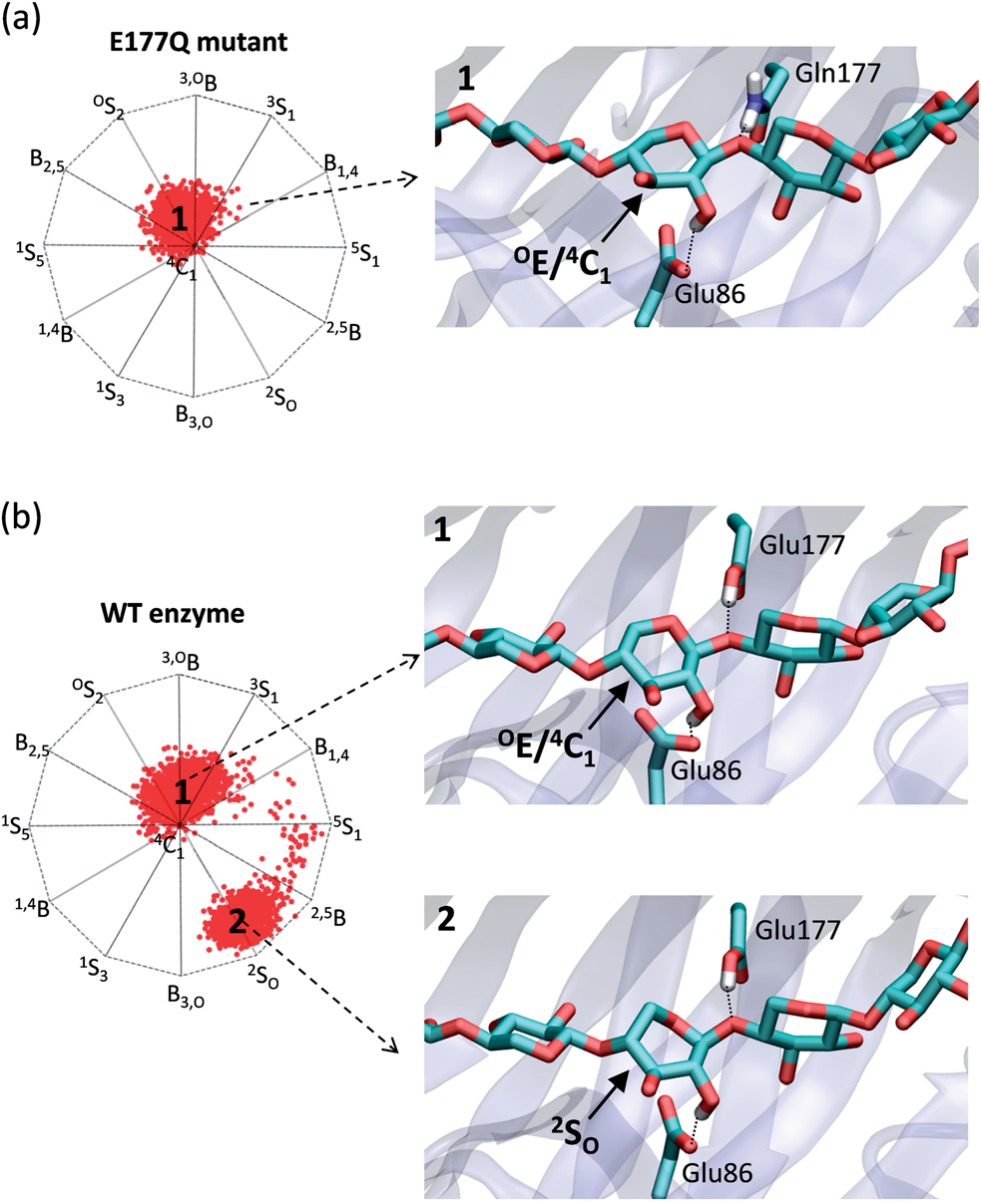
Results of the MD simulations of Michaelis complex of *Trichoderma ressei* GH11 xylanase. The graphs represent the distribution of conformations for each simulation. (a) Mutant enzyme. (b) Wild type enzyme.

To test the above hypothesis, we performed classical and *ab initio* QM/MM molecular dynamics simulations on the structure reported by Wan *et al.*,^30^ which corresponds to the complex of the E177Q enzyme mutant with xylohexaose (E177 is the catalytic acid/base residue). To assess the effect of the mutation on the substrate conformation, the simulations were performed on both the mutant and the WT enzyme (in the last case, we manually reverted the Glu → Gln mutation of the acid/base residue). The simulations for the enzyme mutant complex fairly reproduced the experimental conformation of the xylan hexasaccharide: the –1 xylose ring adopts an intermediate ^O^E/^4^C_1_ conformation (see Fig. 7a). However, once the mutation was reverted, the conformation of the –1 xylose ring started oscillating between ^4^C_1_/^O^E and ^2^S_O_ (Fig. 7b), which is precisely the conformation that has been proposed for GH11 and it is supported by our computed FEL for β-xylose. In addition, QM/MM calculations on the WT enzyme complex show that the ^2^S_O_ conformation is energetically more stable than ^4^C_1_/^O^E, whereas the opposite is found for the mutant complex (see ESI† for details). This is probably due to the structure of the modified enzyme not being able to reproduce the hydrogen bond pattern of the WT enzyme. While a neighboring Asn44 interacts with the acid/base residue acting as a hydrogen bond donor in the WT enzyme, the opposite is found in the mutant enzyme (*i.e.* Asn44 acts as a hydrogen bond acceptor, see Fig. S3†). As a control calculation, we considered an opposite case in which the complex obtained with a modified enzyme conforms with our computed FEL. We selected a family 10 modified retaining xylanase from *Streptomyces olivaceoviridis* (PDB code ; 2D24, Table 1), which was crystallized with a natural substrate, as the previously analyzed complex. The simulations show that there are two stable substrate conformations in the active site (^1^S_3_/B_3,O_ and ^4^C_1_/^4^E), with the distorted one (^1^S_3_/B_3,O_) being the most stable (Fig. S4 of the ESI†). Most importantly, both the modified and the wild type enzyme behave similarly with respect to the conformation of the xyloside at the –1 subsite. Therefore, the complex of modified *Streptomyces olivaceoviridis* GH10 with its natural substrate is a good mimic of the WT Michaelis complex (the “true” Michaelis complex), being in the pathway towards the transition state. In contrast, the complex of *Trichoderma ressei* GH11 xylanase with its natural substrate corresponds to a non-productive complex, in which the substrate has been enforced to adopt a different conformation than the one of the true Michaelis complex. The ^4^C_1_/^O^E conformation observed in this complex cannot be taken as informative of conformational itinerary.

**Fig. 8:**
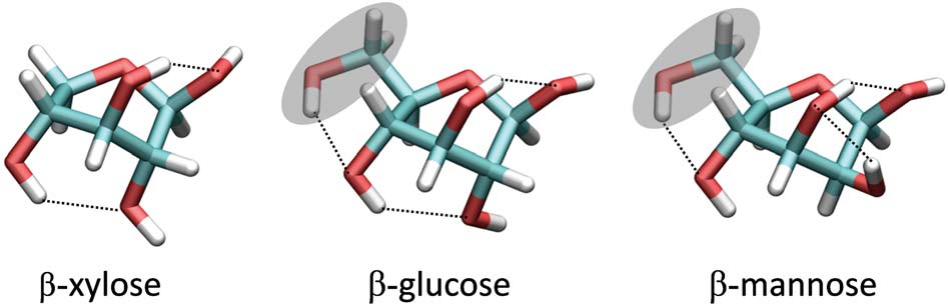
Comparison between ^O^S_2_ conformation of β-xylose, β-glucose and β-mannose (only one of the possible rotameric states of the exocyclic groups is shown).

To analyze how different sugars stabilize different distorted conformations, thus favoring different catalytic itineraries, we compared the computed FEL of β-xylose with the ones previously reported for β-glucose and β-mannose (Fig. 6b and c, respectively). In all cases, only the Northern hemisphere of the Cremer–Pople sphere (Fig. 1) is mechanistically relevant, as found in all experimental structures of β-xylosidases, β-glucosidases and β-mannosidases. Therefore, only the Northern hemisphere is shown in Fig. 6. The landscapes of β-xylose and β-glucose share common features. In both cases, the lowest energy region is in the bottom part of the diagram. However, whereas β-glucose exhibits a second low energy region in the Northwestern quadrant (around B_2,5_), β-xylose contains high energy conformers in this region. These differences can be traced back to changes in sugar ring substitutions. Xylose differs from glucose (and mannose) in the absence of the hydroxymethyl (CH_2_OH) substituent. In glucose and mannose, the CH_2_OH forms intramolecular hydrogen bonds in conformations in the vicinity of B_2,5_ (Fig. 8), something that is not possible in β-xylose. The stability of the Northwestern quadrant is even more pronounced for β-mannose (Fig. 6c) compared to β-glucose and β-xylose. This is due to the particular orientation of the 2-OH substituent in β-mannose (*trans* with respect to H-5, preventing steric interaction between the two) compared to β-glucose and β-xylose (*cis* with respect to H-5). Similarly, the Southeastern quadrant of mannose is higher in energy compared to β-xylose and β-glucose due to unfavorable interactions between the C2 exocyclic group (2-OH) and the hydroxymethyl (CH_2_OH) substituent.

**Fig. 9:**
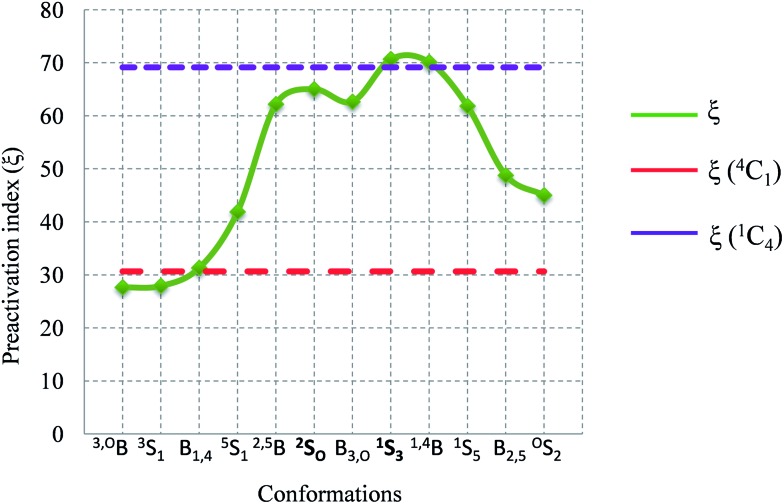
Variation of the values of the preactivation index *ξ* as a function of the ring conformation.

Previous studies of GH conformational maps have revealed that properties other than the free energy alone,^48^ such as the partial charges of C1, O1, and O5, the C1–O1 and C1–O5 bond distances and the orientation of the C1–O1 bond (axial or equatorial, Ω), together with the relative free energy, should be taken into account to identify conformations that are preactivated for catalysis. Following a similar procedure as in ref. 48, we computed all these properties for a number of optimized structures (350) taken from the metadynamics simulation and combined them into a unique index (*ξ*) (see ESI,† pages S6–S12). This index should reflect the likelihood that a given sugar conformation is present in the Michaelis complex of a β-xylosidase, *i.e.* the best preactivated conformation for catalysis. The calculations show that conformations ^2^S_O_ and ^1^S_3_ have high *ξ* values (Fig. 9), whereas ^O^S_2_, ^4^C_1_, ^3,O^B and ^3^S_1_ have particularly low *ξ* indexes. These results again support ^1^S_3_ → [^4^H_3_]^ǂ^ → ^4^C_1_ and ^2^S_O_ → [^2,5^B]^ǂ^ → ^5^S_1_, but not ^O^E → [^O^S_2_]^ǂ^ → B_2,5_, as possible itineraries for hydrolysis reactions catalyzed by β-xylosidases.

## Summary and conclusions

In this work, we have computed the conformational free energy landscape for cyclohexane and β-xylose. The structure and relative free energy of the eight energy minima of cyclohexane (two chair structures, ^4^C_1_ and ^1^C_4_, which are the global minima, and six skew-boat structures) and their interconversion energy barriers agree well with the experimental information available. Interestingly, we found out that the structure of the TS between the chair and the skew-boat conformers deviates by 9.2° in the *θ* puckering coordinate with respect to a “pure” half-chair conformer (*θ* of 50.8° and 129.2°), which exhibits four consecutive coplanar atoms. Our simulations also show that, contrary to the conclusions of a force-field-based metadynamics investigation,^37^ the free energy landscape remains unaltered by the use of polar or Cartesian collective variables. As already reported in previous *ab initio* metadynamics simulations of pyranoses,^15^ but not reproduced in the above force-field-based study,^37^ the puckering amplitude sphere is not spherical but elliptical, with a smaller radius at the poles compared to the equator.

The conformational FEL of β-xylose contains two local minima, corresponding to the two chair conformers (^4^C_1_ and ^1^C_4_), as well as wide minimum in between B_3,O_ and ^O^S_2_. As previously found for other pyranoses, the puckering coordinates of all the –1 xylose saccharides extracted from experimental structures of β-xylosidase Michaelis complexes are located, without exceptions, in low-energy regions of the FEL. This finding, which is likely to be general in GH complexes, suggests that the factors governing the distortions present in these complexes are dictated by the intrinsic properties of a single saccharide unit (β-glucose in β-glucosidases,^43^ β-mannose in β-mannosidases,^48^ α-mannose for α-mannosidases,^15^ α-fucose for α-fucosidases^70^ and β-xylose for β-xylosidases) and that enzyme–substrate interactions have evolved to fulfill all those criteria for efficient catalysis.

Two itineraries, ^1^S_3_ → [^4^H_3_]^ǂ^ → ^4^C_1_ and ^2^S_O_ → [^2,5^B]^ǂ^ → ^5^S_1_, which pass through low energy regions of the FEL (Fig. 6a), are consistent with our simulations. Conformations ^2^S_O_ and ^1^S_3_ are preactivated for catalysis in terms of free energy, anomeric charge and bond distances (Fig. 9). However, the ^O^E → [^O^S_2_]^ǂ^ → B_2,5_ itinerary, recently proposed for *Trichoderma ressei* GH11 xylanase, is not compatible with the FEL: conformations around ^O^E are not preactivated for catalysis and the ^O^E → [^O^S_2_]^ǂ^ → B_2,5_ route passes through a high energy region of the FEL. Additional classical and QM/MM molecular dynamics simulations on the complex of a xylose hexasaccharide with *Trichoderma ressei* GH11 xylanase show that the ^4^C_1_/^O^E conformation observed in the reported enzyme complex is due to enzyme mutation. Although this cannot be taken as general (similar calculations on the complex of modified *Streptomyces olivaceoviridis* GH10 with its natural substrate show that it is a good mimic of the true Michaelis complex), it indicates that complexes obtained from modified enzymes do not necessarily represent the structures that take place in the reaction coordinate.

In summary, the simulations of the FEL of β-xylose reveal lower free energy regions that agree with two of the three conformational routes previously proposed for different β-xylosidase families. We also show that the preactivation index is a good measure of the general preferences of β-xylosidases to accommodate specific β-xylose conformations at the –1 enzyme subsite. Most likely, the higher flexibility of the xylose unit in comparison with other pyranoses, due to the lack of the hydrogen-bond interactions with the CH_2_OH group, can cause the xylose ring to adopt different conformations (^2^S_O_ and ^1^S_3_) with small changes of the active site structure, as encountered in different xylanase families.
