# Supplementary material for: The complete conformational free energy landscape of β-xylose reveals a two-fold catalytic itinerary for β-xylanases
Source: Chem Sci. 2014 Oct 27;6(2):1167–77. doi: 10.1039/c4sc02240h (PMC5811086; doi:10.1039/c4sc02240h)
Supplement: Supplementary file 1 [file SC-006-C4SC02240H-s001.pdf]

Electronic Supplementary Material (ESI) for Chemical Science.
This journal is © The Royal Society of Chemistry 2014

Supporting Information

The complete conformational free-energy landscape of *β*-xylose reveals a two-fold catalytic itinerary for *β*-xylanases

Javier Iglesias-Fernández,[1] Lluís Raich,[1] Albert Ardèvol[2] and Carme Rovira[1,3,*]

[1] Departament de Química Orgànica and Institut de Química Teòrica i Computacional (IQTCUB), Universitat de Barcelona, Martí i Franquès 1, 08028 Barcelona, Spain.

[2] Department of Chemistry and Applied Biosciences, ETH Zürich, USI Campus, 6900 Lugano, Switzerland.

[3] Institució Catalana de Recerca i Estudis Avançats (ICREA), Passeig Lluís Companys, 23, 08018 Barcelona, Spain.

CONTENTS

1. Transition state between skew-boat and chair local minima in cyclohexane

2. Analysis of the projected puckered vector

3. Modeling of the Michaelis complex of *Trichoderma ressei* GH11 xylanase

4. Modeling of the Michaelis complex of *Streptomyces olivaceoviridis* GH10 xylanase

5. Analysis of β-xylose conformations (degree of preactivation of each conformation)

1. Transition state between skew-boat and chair local minima in cyclohexane

As described in the main text, the transition states between skew-boat and chair conformers of cyclohexane occur at θ = 60°, 120°, thus they deviate from pure half-chair conformations (θ = 50.8°, 129.2°) by 9.2°. To assess whether the potential energy surface (PES) also reflects this deviation, we performed a standard transition state search using the GAUSSIAN 09 package[1] and the PBE/6-311++G**, M062X/6-311++G** and MP2/6-311++G** levels of theory, starting from the TS obtained from the metadynamics simulation. The corresponding TS was found at θ = 62°, 118° (PBE), 61°, 119° (M062X) and 61°, 119° (MP2), in agreement of recent calculations by Stortz.[2] Therefore, both the PES and FES (metadynamics) show a similar variation from a pure half-chair conformer.

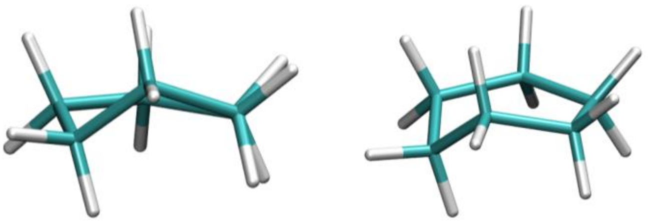

Figure S1. Transition state for interconversion between the skew-boat and chair conformer of cyclohexane computed at the MP2/6-311++G** level of theory.

2. Analysis of the projected puckered vector

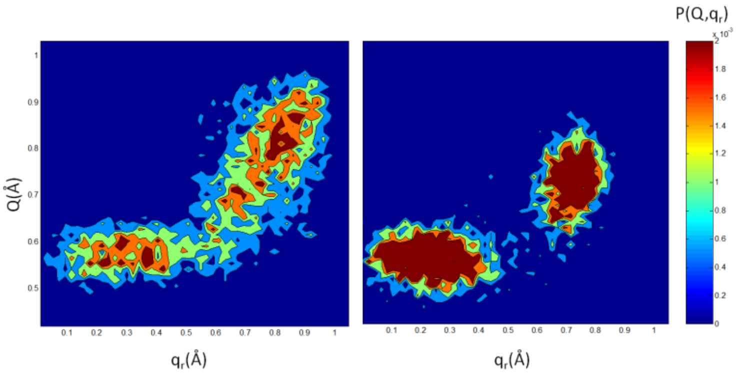

Figure S2. Projection of the $q_r = \sqrt{q_x^2 + q_y^2}$ vs the total puckering amplitude Q during the q_x/q_y (left) and θ/ϕ (right) metadynamics simulations.

3. Modeling of the Michaelis complex of *Trichoderma ressei* GH11 xylanase

Computational details: The initial structure for the simulations was taken from the recently reported structure of *Trichoderma ressei* GH11 xylanase in complex with a xyloglucan hexasaccharide (PDB: 4HK8). In this structure, the acid/base Glu residue is mutated to glutamine (Glu177Gln) and the xylose saccharide at the -1 subsite adopts a $^{4}C_{1}/^{O}E$ conformation. Two separate systems were modeled, with and without the mutation of the acid/base residue (in the last case, the mutation was manually reverted). For each system, two simulations were performed, one with the sugar initially in a $^{4}C_{1}/^{O}E$ conformation and the other with the sugar in the $^{2}S_{O}$ conformation (the last one was obtained by a restrained relaxation). The protonation states and hydrogen atom positions of all amino acid residues were taken from the crystal structure, except His155, which was changed from double to single protonation due to the close contact with Ser139. All crystallographic water molecules were retained and extra water molecules were added to form a 15 Å water box around the protein surface. Five chloride ions were also added to neutralize the enzyme charge.

Molecular dynamics (MD) simulations using Amber11 software[1] were performed. The protein was modeled with the FF99SB force-field,[3] the carbohydrate substrate with the GLYCAM06 force-field[5] and water molecules were described with the TIP3P force field.[6] The MD simulations were carried out in several steps. First, the systems were minimized, keeping the protein and substrate fixed. Then, the entire systems were allowed to relax. To gradually reach the desired temperature of 300 K in the MD simulations, weak spatial constraints were initially added to the protein and substrate, while the water molecules and chloride ions were allowed to move freely at 100K. The constraints were then removed and the working temperature of 300 K was reached after two more 100K heatings in the NVT ensemble. Afterwards, densities were converged up to water density at 300K in the NPT ensemble. The simulations were further extended to 18 ns, when equilibration was reached. In the case of the simulations of the wild type (WT) enzyme, the acid/base residue (Glu177) was restrained (after reverting the Gly → Gln mutation) for the first 15 ns. Analysis of the trajectories was carried out using standard tools of AMBER and VMD.[7] A snapshot of each MD-equilibrated structure was taken for the subsequent QM/MM calculations.

QM/MM calculations were performed using the method developed by Laio *et al.*,[8] which combines Car–Parrinello MD,[9] based on Density Functional Theory (DFT),

with force-field MD methodology. In this approach, the system is partitioned into quantum mechanics (QM) and molecular mechanics (MM) fragments. The dynamics of the atoms on the QM fragment depend on the electronic density, ρ(r), computed with Density Functional Theory, whereas the dynamics of the atoms on the MM fragment is ruled by an empirical force field. The QM/MM interface is modeled by the use of link-atom that saturates the QM region. The electrostatic interactions between the QM and MM regions were handled via a fully Hamiltonian coupling scheme,[8] where the short-range electrostatic interactions between the QM and the MM regions are explicitly taken into account for all atoms. An appropriately modified Coulomb potential was used to ensure that no unphysical escape of the electronic density from the QM to the MM region occurs. The electrostatic interactions with the more distant MM atoms were treated via a multipole expansion. Bonded and van der Waals interactions between the QM and the MM regions were treated with the standard AMBER force field. Long-range electrostatic interactions between MM atoms were described with the P3M implementation,[10] using a 64 x 64 x 64 $Å^3$ mesh. The QM region included the xylose rings at the -1 and +1 subsites, half rings of the saccharides at the -2 and +2 subsites and the acid/base residue, leading to a total number of 66/67 QM atoms (for the WT/mutated forms of the enzyme) and 40346 MM atoms. The QM region was enclosed in an isolated supercell of size 22.6 x 21.2 x 16.9 $Å^3$ (WT enzyme) and 13.3 x 24.5 x 18.3 $Å^3$ (mutated enzyme). Kohn–Sham orbitals were expanded in a planewave basis set with a kinetic energy cutoff of 70 Ry. Norm-conserving Troullier–Martins *ab initio* pseudopotentials[11] were used for all elements. The calculations were performed using the Perdew, Burke and Ernzerhoff generalized gradient-corrected approximation (PBE).[12] This functional form has been proven to give a good performance in the description of hydrogen bonds[13] and was already used with success in previous works on glycoside hydrolases and transferases.[14]

Results: The simulation of the modified enzyme showed that the -1 saccharide keeps the $^4C_1$ conformation during the entire classical MD simulation (Figure S3, top). Even starting from a ^2S_O conformation (enforced by geometric restraints), the sugar rapidly evolved towards the $^4C_1$ conformation. In the case of the WT enzyme, the conformation of the sugar oscillates between ^2S_O and $^4C_1$. Therefore, although the xylose ring adopts solely the $^4C_1$ conformation in the mutated enzyme, two conformations ($^4C_1$ and ^2S_O) are possible in the WT enzyme, evidencing that the Glu177Gln mutation prevents the system from adopting the highly populated ^2S_O conformation found in the WT enzyme.

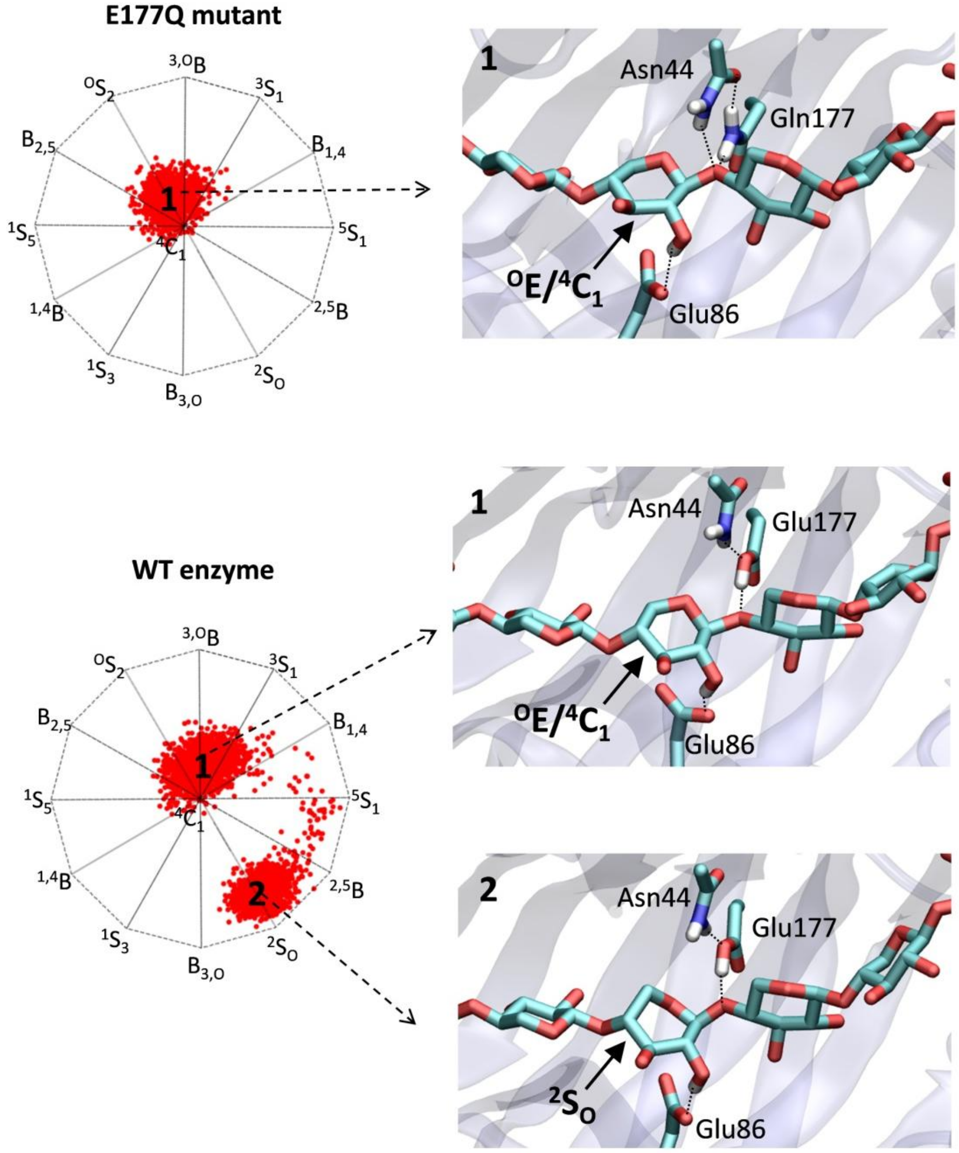

Figure S3. Results of the MD simulations of Michaelis complex of *Trichoderma ressei* GH11 xylanase. The graphs represent the distribution of conformations for each simulation.

To elucidate the most favored conformation, QM/MM calculations were performed starting from a snapshot of the classical MD simulations. Four systems were considered: WT-$^{4}C_1$, WT-$^{2}S_O$, Mut-$^{4}C_1$ and Mut-$^{2}S_O$ (Mut = mutated enzyme). Each system was equilibrated by QM/MM MD for a period of 2ps, followed by QM/MM MD simulated annealing and structure minimization. The results obtained show that the $^{4}C_1$ is the most stable conformer for the mutated protein (Mut-$^{4}C_1$ is lower in energy than Mut-$^{2}S_O$ by

6.7 kcal/mol) but $^{2}S_{O}$ becomes the most stable form for the WT enzyme (WT-$^{2}S_{O}$ is lower in energy than WT-$^{4}C_{1}$ by 2.1 kcal/mol).

4. Modeling of the Michaelis complex of *Streptomyces olivaceoviridis* GH10 xylanase

Computational details: The initial structure for the simulations was taken from the reported structure of *Streptomyces olivaceoviridis* GH10 xylanase in complex with a xyloglucan pentasaccharide (PDB: 2D24). The structure is a double mutant of residues 127 and 128 (N127S and E128H, being 128 the acid/base residue) and the xylose saccharide at the -1 subsite adopts a $^{1}S_{3}$ conformation. Similarly to the latest case, two separate systems were modeled, with and without the mutations. The mutated and the missing residues (304 to 312) were taken from the structure of PDB entry 1ISV. The protonation states of all amino acid residues were taken according to protein environment. All crystallographic water molecules were retained and extra water molecules were added to form a 15 Å water box around the protein surface. One chloride ion was also added to neutralize the enzyme charge. MD simulations using the Amber11 software were performed 20-30 ns (until equilibration of the protein backbone RMSD was reached) followed by 2 ps of QM/MM MD simulation, according to the procedure detailed in the previous section. The QM region included the xylose rings at the -1 and -2 subsites, the half ring of the +3 sugar and residue 128, leading to a total number of 59/60 QM atoms and 82930/82918 MM atoms (for the WT/mutated enzyme forms). The QM region was enclosed in an isolated supercell of size 18.1 x 15.7 x 20.5 $Å^3$ (WT enzyme) and 14.1 x 19.5 x 19.9 $Å^3$ (modified enzyme).

Results: In contrast to the results obtained for *Trichoderma ressei* GH11 xylanase (previous system), *Streptomyces olivaceoviridis* GH10 xylanase shows the same puckering distribution pattern either with or without the mutations (Figure S4). Specifically, the ring conformation oscillates between $^{4}C_{1}$/^{4}E and $^{1}S_{3}$/$B_{3,O}$ in both the WT and the modified enzyme. Analysis of the MD trajectories suggests that the change of conformation is related to the presence/absence of hydrogen bond interaction between residue 128 and the glycosidic oxygen, although further work using free energy methods is necessary to establish the rules that govern the conformational transition. QM/MM MD simulations show that in both cases $^{1}S_{3}$/$B_{3,O}$ is the most stable conformer, with the difference being more pronounced in the wild type form than in the modified enzyme (8.3 kcal/mol vs 0.3 kcal/mol, respectively).

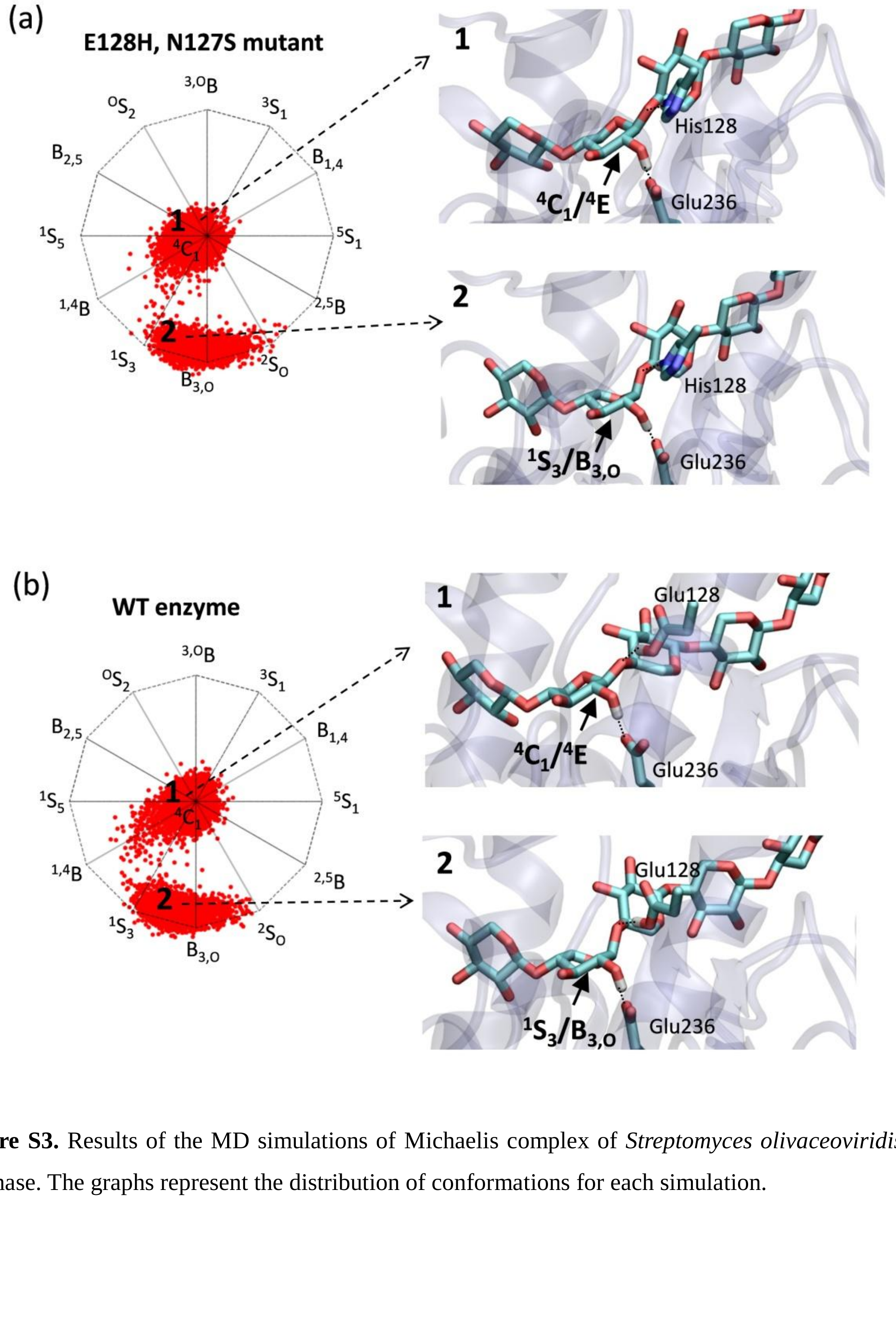

Figure S3. Results of the MD simulations of Michaelis complex of *Streptomyces olivaceoviridis* GH10 xylanase. The graphs represent the distribution of conformations for each simulation.

5. Analysis of β-xylose conformations (degree of preactivation of each conformation)

Apart from the relative energy of each conformation, other relevant properties (structural and electronic) should be taken into account to determine which conformations are the best preactivated for catalysis. From a structural point of view, the two most relevant parameters are the C1-O5 and C1-O1 distances, as these are the ones that change during catalysis (namely, the C1-O5 acquires partial double bond character and the C1-O1 distance lengthens in the oxocarbenium ion-like transition state[15,16]). The orientation of the C1-O1 bond (axial for an oxocarbenium ion) is another structural factor to be taken into account. From an electronic point of view, the most relevant parameters are the relative charge at the anomeric carbon (q_{C1}), ring oxygen (q_{O5}) and glycosidic oxygen (q_{O1}). In our previous work in β-glucose[16], we showed that some conformers ($^{1,4}B$, $^{1}S_{3}$, $B_{3,O}$, $^{2}S_{O}$) fulfill the maximum number of energetic, structural and electronic requirements for efficient catalysis, namely: they have a low free energy, large C1-O1 / C1-O5 ratio, large q_{C1} and axial C1-O1. To track these changes, a set of 300 structures were selected from the metadynamics simulation of β-xylose and submitted to geometry optimization. The optimized structures were clustered to the canonical distortions according to their θ and φ final values and their distances and charges were analyzed, as well as the orientation of C1-O1 bond (measured as the angle between the bond of interest and the average ring plane, $\Phi_{C1\text{-}O1}$). Each of the six parameters was computed for each structure within the group, and the average values were assigned to the corresponding canonical conformation. The corresponding values for the undistorted $^{4}C_{1}$ structure were obtained from a separate equilibrium simulation starting from this conformation.

Figure S4 shows that the C1-O1 and C1-O5 bond distances display significant variations (up to 0.03 Å) with ring conformation. Distortions in the range from $^{2,5}B$ to $^{1}S_{5}$ and the $^{1}C_{4}$ conformation show the major increase in C1-O1 distance and decrease in C1-O5 distance, with respect to an undistorted $^{4}C_{1}$ conformation (broken lines). Therefore, these conformations are the ones that most resemble the TS of the hydrolysis reaction in terms of bond distances. The variations in the atomic (ESP) charges for the atoms C1, O1 and O5 (Figure S5) show that the conformations having a larger q_{C1} are the ones in the range between $^{1}S_{5}$ and $^{1,4}B$ and the $^{O}S_{2}$ conformation. Only slightly changes are observed on the values of q_{O1} and q_{O5}. The orientation of the C1-O1 bond

was analyzed by measuring the angle Ω between the bond and the average plane of the ring (Figure S6). For the C1-O1 bond, which presents positive values for this parameter due to the β-sugar configuration, increasing positive values indicate a high tendency to an axial configuration which favors catalysis. As shown in Figure S6, conformations around $^{1,4}B$ show the best bond orientation.

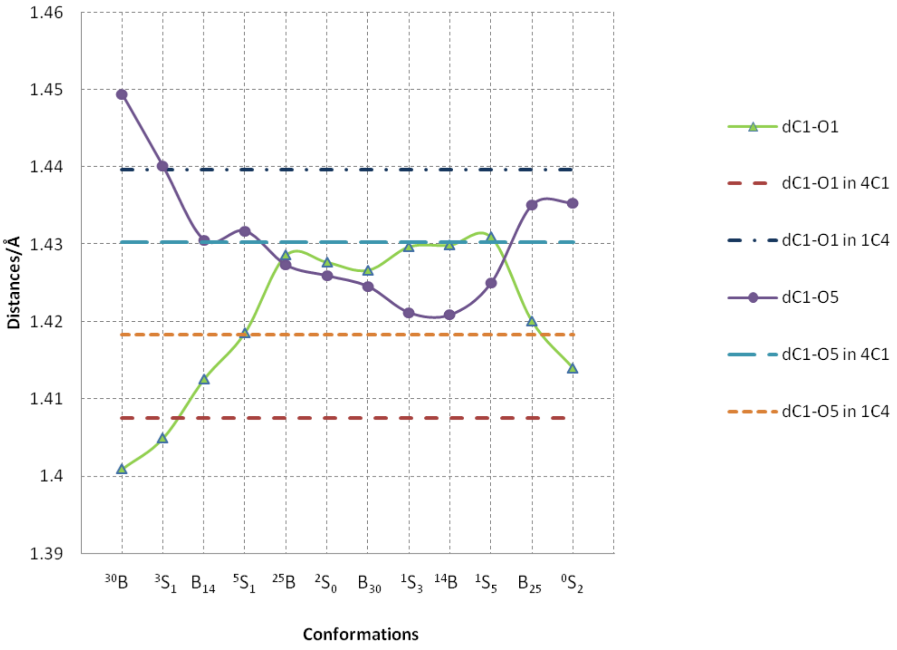

Figure S4. Structural changes associated with ring distortion.

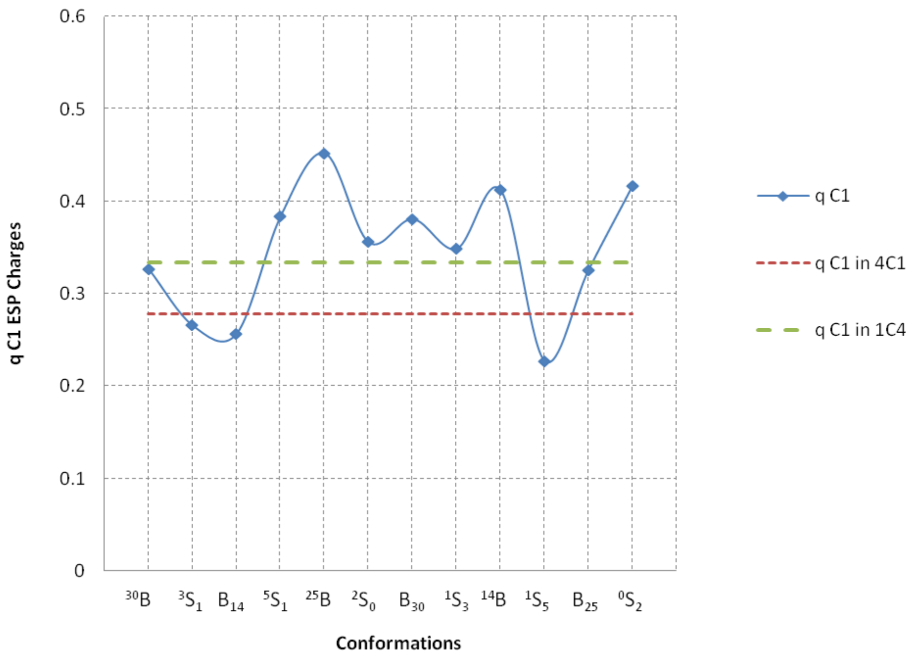

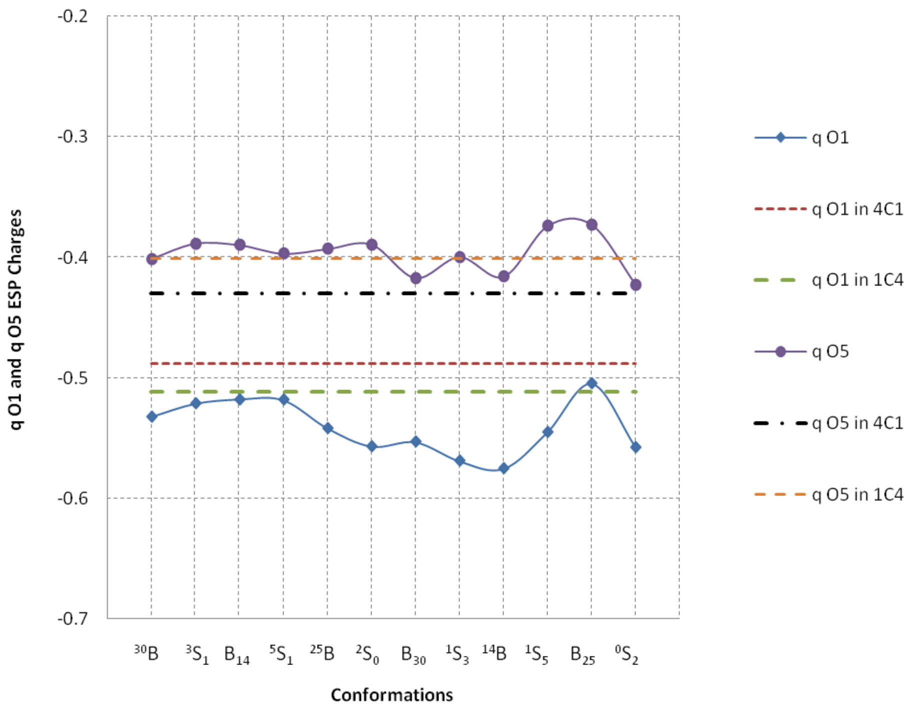

Figure S5. Electronic rearrangements associated with ring distortion. Electronic charges are given in electron units.

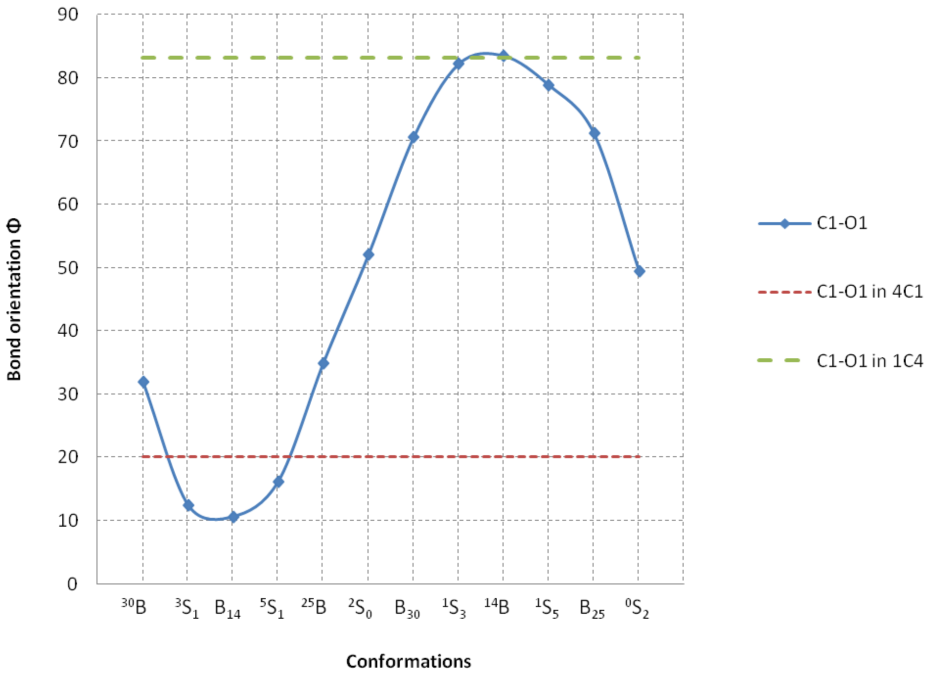

Figure S6. Bond orientation (axial or equatorial) with respect to the mean plane formed of the sugar molecule. Angles are given in degrees.

Similarly to what was previously found for α,β-mannose,[14e,17] there are no conformers with optimum values of all parameters determining the degree of preactivation. Therefore, conclusions cannot be based on one parameter separately but all of them need to be considered together. Therefore, we combined all of the above parameters (q_{C1}, q_{O5}, q_{O1}, $d_{C1\text{-}O1}$, $d_{C1\text{-}O5}$ and $\Phi_{C1\text{-}O1}$) along with the relative free energy (ΔG_{rel}) into a unique index that could reflect the likelihood that a given conformation would be adopted in the Michaelis complex of a β-xylosidase. This was done by assigning for each conformation j a score for each parameter x_i using the following formulas:

$$\mathrm{score}\left(x_{i,j}\right) = \frac{x_{i,j} - x_{i,j}^{min}}{x_{i,j}^{max} - x_{i,j}^{min}} \times 100 \quad \text{for } x_i = d_{C1\text{-}O1}, q_{C1}, q_{O1}, \left|\Phi_{C1-O1}\right|$$

$$\mathrm{score}\left(x_{i,j}\right) = \frac{x_{i,j}^{max} - x_{i,j}}{x_{i,j}^{max} - x_{i,j}^{min}} \times 100 \quad \text{for } x_i = d_{C1\text{-}O5}, q_{O5}, \Delta G_{rel}$$

The values of the parameters and the corresponding scores are given in Table S1. Since the score for each parameter is normalized, the scores can be directly compared. We then defined an index ξ_j as the average of the scores for the n parameters (n = 7 in our case) for a given conformation j:

$$\xi_j = \sum_i \text{score}\left(x_{i,j}\right) / n$$

In this formulation, the conformations displaying the highest values of ξ are the most likely candidates to be the MC. Figure S7 shows the variation of the ξ with ring distortion. There is no single conformation with the optimum values for every parameter (ξ = 100). The highest values of ξ occur for conformations on the center of the diagram ($^{2}S_{O}$, $^{1}S_{3}$,$^{1,4}B$) and the $^{1}C_{4}$ conformation.

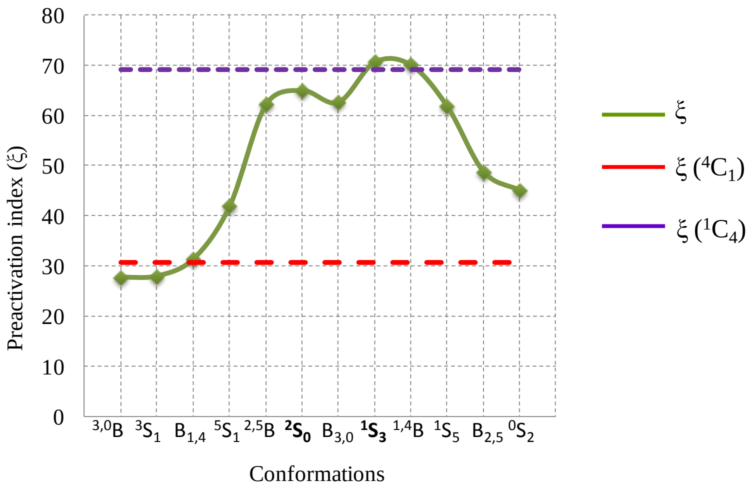

Figure S7. Preactivation index (ξ) for each canonical conformation.

Table S1. Values of the different properties of interest, along with its score (in grey) and the resulting preactivation index (ξ), associated to each canonical conformation. Distances are given in angstroms, ESP charges in electrons, $\Phi_{C1\text{-}O1}$ in degrees and free energy (ΔG_{rel}) in kcal/mol.

Conformation	q(C1)	q (O1)	q (O5)	d(C1-O1)	d(C1-O5)	$\Phi_{C1\text{-}O1}$	ΔG_{rel}	ξ
^{30}B	0.33	-0.53	-0.40	1.401	1.449	31.94	8.13	28
	44	51	50	0	0	29	19	
$^{3}S_{1}$	0.27	-0.52	-0.39	1.405	1.440	12.42	7.56	28
	17	38	72	10	30	2	25	
B_{14}	0.26	-0.52	-0.39	1.413	1.431	10.61	8.96	31
	13	34	70	30	61	0	11	
$^{5}S_{1}$	0.38	-0.52	-0.40	1.419	1.432	16.19	7.95	42
	70	35	58	46	57	8	21	
^{25}B	0.45	-0.54	-0.39	1.429	1.427	34.90	6.69	62
	100	61	65	71	71	33	34	
$^{2}S_{0}$	0.36	-0.56	-0.39	1.428	1.426	52.04	5.35	65
	57	79	71	69	76	57	47	
B_{30}	0.38	-0.55	-0.42	1.427	1.425	70.59	5.53	63
	68	75	22	66	80	82	45	
$^{1}S_{3}$	0.35	-0.57	-0.40	1.430	1.421	82.24	6.86	71
	54	93	53	74	91	98	32	
^{14}B	0.41	-0.58	-0.42	1.430	1.421	83.52	8.26	70
	82	100	24	75	92	100	18	
$^{1}S_{5}$	0.23	-0.54	-0.37	1.431	1.425	78.90	8.06	62
	0	65	98	77	79	94	20	
B_{25}	0.33	-0.50	-0.37	1.420	1.435	71.24	10.08	49
	44	19	100	49	46	83	0	
$^{0}S_{2}$	0.42	-0.56	-0.42	1.414	1.435	49.44	9.40	45
	84	80	12	34	45	53	7	

**$^{1}C_{4}$**	0.33	-0.51	-0.40	1.440	1.418	83.29	4.00	69
	47	26	50	100	100	100	60	
**$^{4}C_{1}$**	0.28	-0.49	-0.43	1.408	1.430	20.20	0.00	31
	23	0	0	17	62	13	100	

References

(1) Gaussian 09, Revision **D.01**, M. J. Frisch, G. W. Trucks, H. B. Schlegel, G. E. Scuseria, M. A. Robb, J. R. Cheeseman, G. Scalmani, V. Barone, B. Mennucci, G. A. Petersson, H. Nakatsuji, M. Caricato, X. Li, H. P. Hratchian, A. F. Izmaylov, J. Bloino, G. Zheng, J. L. Sonnenberg, M. Hada, M. Ehara, K. Toyota, R. Fukuda, J. Hasegawa, M. Ishida, T. Nakajima, Y. Honda, O. Kitao, H. Nakai, T. Vreven, J. A. Montgomery, Jr., J. E. Peralta, F. Ogliaro, M. Bearpark, J. J. Heyd, E. Brothers, K. N. Kudin, V. N. Staroverov, R. Kobayashi, J. Normand, K. Raghavachari, A. Rendell, J. C. Burant, S. S. Iyengar, J. Tomasi, M. Cossi, N. Rega, J. M. Millam, M. Klene, J. E. Knox, J. B. Cross, V. Bakken, C. Adamo, J. Jaramillo, R. Gomperts, R. E. Stratmann, O. Yazyev, A. J. Austin, R. Cammi, C. Pomelli, J. W. Ochterski, R. L. Martin, K. Morokuma, V. G. Zakrzewski, G. A. Voth, P. Salvador, J. J. Dannenberg, S. Dapprich, A. D. Daniels, Ö. Farkas, J. B. Foresman, J. V. Ortiz, J. Cioslowski, and D. J. Fox, Gaussian, Inc., Wallingford CT, 2009.

(2) Stortz, C. A. *J. Phys. Org. Chem.*, 2010, **23**, 1173-1186.

(3) Pearlman, D. A.; Case, D. A.; Caldwell, J. W.; Ross, W. S.; Cheatham, T. E.; Debolt, S.; Ferguson, D.; Seibel, G.; Kollman, P. *Comp. Phys. Commun.* **1995**, *91*, 1-41.

(4) Sorin, E. J.; Pande, V. S. *Biophys. J.* **2005**, *88*, 2472-2493.

(5) Kirschner, K. N.; Yongye, A. B.; Tschampel, S. M.; Gonzalez-Outeirino, J.; Daniels, C. R.; Foley, B. L.; Woods, R. J. *J. Comput. Chem.* **2008**, *29*, 622-655.

(6) Jorgensen, W. L.; Chandrasekhar, J.; Madura, J. D.; Impey, R. W.; Klein, M. L. *J. Chem. Phys.* **1983**, *79*, 926-935.

(7) Humphrey, W.; Dalke, A.; Schulten, K. *J. Mol. Graph.* **1996**, *14*, 33-&.

(8) Laio, A.; VandeVondele, J.; Rothlisberger, U. *J. Chem. Phys.* **2002**, *116*, 6941-6947.

(9) Car, R.; Parrinello, M. *Phys. Rev. Lett.* **1985**, *55*, 2471-2474.

(10) Hunenberger, P. H. *J. Chem. Phys.* **2000**, *113*, 10464-10476.

(11) Troullier, N.; Martins, J. L. *Phys. Rev. B.* **1991**, *43*, 1993-2006.

(12) Perdew, J. P.; Burke, K.; Ernzerhof, M. *Phys. Rev. Lett.* **1996**, *77*, 3865-3868.

(13) Ireta, J.; Neugebauer, J.; Sheffler, M. *J. Phys. Chem. A* **2004**, *108*, 5692-5698.

(14) (a) Biarnés, X.; Ardèvol, A.; Iglesias-Fernández, J.; Planas, A.; Rovira, C. *J. Am. Chem. Soc.* **2011**, *133*, 20301-20309. (b) Petersen, L.; Ardevol, A.; Rovira, C.; Reilly, P. J. *J. Phys. Chem. B.* **2009**, *113*, 7331-7339. (c) Ardèvol, A.; Rovira, C. *Angew. Chem. Int. Ed. Engl.* **2011**, *50*, 10897-10901. (d) Rojas-Cervellera, V.; Ardevol, A.; Boero, M.; Planas, A.; Rovira, C. *Chemistry.* **2013**, *19*, 14018-14023. (e) Thompson, A. J.; Dabin, J.; Iglesias-Fernandez, J.; Ardevol, A.; Dinev, Z.; Williams, S. J.; Bande, O.; Siriwardena, A.; Moreland, C.; Hu, T. C.; Smith, D. K.; Gilbert, H. J.; Rovira, C.; Davies, G. J. *Angew. Chem. Int. Ed. Engl.* **2012**, *51*, 10997-11001.

(15) Whitfield, D. M. *Carbohydr. Res.* **2007**, *342*, 1726-1740.

(16) Biarnes, X.; Ardevol, A.; Planas, A.; Rovira, C.; Laio, A.; Parrinello, M. *J. Am. Chem. Soc.* **2007**, *129*, 10686-10693.

(17) Ardevol, A.; Biarnes, X.; Planas, A.; Rovira, C. *J. Am. Chem. Soc.* **2010**, *132*, 16058-16065.
